# Supplementary material for: Psychometric evaluation of the Chinese version of Risky Loot Box Index (RLI) and cross-sectional investigation among gamers of China
Source: PeerJ. 2025 Mar 25;13:e19164. doi: 10.7717/peerj.19164 (PMC11952041; doi:10.7717/peerj.19164)
Supplement: Supplemental Information 3 [file peerj-13-19164-s003.docx]

**The Chinese version of RLI**

| Items | 条目 |
| --- | --- |
| 1. I frequently play games longer than I intend to, so I can earn Loot Boxes. | 1.为了（通过游戏）赚取战利品箱，我玩游戏的时间经常比预期长。 |
| 2.I believe obtaining items from Loot Boxes is an effective way to generate money. | 2.我认为从战利品箱中获得物品是一种有效的赚钱方法。 |
| 3.I will play for long periods of time to earn Loot Boxes. | 3.为了（通过游戏）赚取战利品箱，我经常会玩很长时间游戏。 |
| 4.Receiving items from Loot Boxes is a primary reason why I play video games. | 4.从战利品箱中获得道具/角色是我玩电子游戏的主要原因。 |
| 5.I buy Loot Boxes with the hope of receiving valuable items to sell. | 5.我购买战利品箱是希望能获得有价值的道具/角色进行出售。 |
| 6.I have put off other activities, work, or chores to be able to earn or buy more Loot Boxes. | 6.为了能够赚取或者购买更多的战利品箱，我曾经推迟过其他活动、工作或家务。 |
| 7.Once I open a Loot Box, I often feel compelled to open another. | 7.一旦打开了一个战利品箱，我经常会忍不住打开另一个。 |
| 8.I have sometimes spent more on Loot Boxes than I could afford. | 8.有时我在战利品箱上花的钱超出个人承受能力。 |
| 9.I have bought more Loot Boxes after failing to receive valuable items. | 9.在没有获得有价值的道具/角色后，我购买了更多的战利品箱。 |

***Note:*** 5-point Likert scale, ranging from "完全同意" (Strongly Agree) to "完全不同意" (Strongly Disagree)
