# Supplementary material for: Psychometric evaluation of the Chinese version of Risky Loot Box Index (RLI) and cross-sectional investigation among gamers of China
Source: PeerJ. 2025 Mar 25;13:e19164. doi: 10.7717/peerj.19164 (PMC11952041; doi:10.7717/peerj.19164)
Supplement: Supplemental Information 4 [file peerj-13-19164-s004.pdf]

STROBE Statement—checklist of items that should be included in reports of observational studies

	Item No.	Recommendation	Page No.	Relevant text from manuscript
**Title and abstract**	1	(*a*) Indicate the study's design with a commonly used term in the title or the abstract	1	Psychometric evaluation of the Chinese version of risky loot box index (RLI) and cross-sectional investigation among gamers of China
		(*b*) Provide in the abstract an informative and balanced summary of what was done and what was found	2	The study translated the RLI into a Chinese version and conducted a psychometric evaluation, and investigated the current use of loot boxes by Chinese gamers. We found that the use of loot boxes was significantly associated with both gaming and gambling, as well as negative emotions. Similar to gambling, loot boxes can lead to property damage.
**Introduction**				
Background/rationale	2	Explain the scientific background and rationale for the investigation being reported	3-4	Loot boxes are the most important fee-based component in video games. Studies found that loot boxes share similar characteristics to gambling. In order to better study loot boxes, the RLI has been development and widely used in several countries, while in China, there

				is a lack of a loot-box assessment tool with good psychometric properties.
Objectives	3	State specific objectives, including any prespecified hypotheses	4	we aimed to translate the RLI into the Chinese version. Then, the factor structure, validity, and reliability of RLI-C will be evaluated among Chinese video gamers, and impact factors that related to loot box usage will be explored.
**Methods**				
Study design	4	Present key elements of study design early in the paper	5	The data of this study were collected by an online survey and an offline survey.
Setting	5	Describe the setting, locations, and relevant dates, including periods of recruitment, exposure, follow-up, and data collection	5	The online survey was published on a Chinese online forum called Baidu Tieba. We chose two of the most popular video game sub-forums（“Genshin Impact” and “Counter-Strike”）to distribute the questionnaire. For the offline survey, the players of internet cafes in Changsha (a city in China) were invited to complete this questionnaire.
Participants	6	(*a*) *Cohort study*—Give the eligibility criteria, and the sources and methods of selection of participants. Describe methods of follow-up<br>*Case-control study*—Give the eligibility criteria, and the sources and methods of case ascertainment and control selection. Give the rationale for the choice of cases and controls	5-6	For quality control, the following criteria were used to remove invalid questionnaires: (1) answer time < 120 s; (2)

		Cross-sectional study—Give the eligibility criteria, and the sources and methods of selection of participants		wrong answer to “trap” question, like “the results of 2 plus 3”; (3) same option for ten consecutive questions. Meanwhile, Questionnaires answered: (1) I haven't played any games recently (wasn't a gamer); (2) age＜18 also were excluded.
Variables	7	Clearly define all outcomes, exposures, predictors, potential confounders, and effect modifiers. Give diagnostic criteria, if applicable	5-6	The **Procedure** section describes the demographic information collected for the study and the scales involved.
Data sources/ measurement	8*	For each variable of interest, give sources of data and details of methods of assessment (measurement). Describe comparability of assessment methods if there is more than one group	6-8	The **Measures** section describes in detail how the individual variables are acquired
Bias	9	Describe any efforts to address potential sources of bias	7, 12	The same web-based questionnaire was used for both samples and both were completed through online responses to avoid bias. Meanwhile, the study provides a detailed description of gambling and loot boxes to prevent comprehension bias
Study size	10	Explain how the study size was arrived at	9	The sample size of EFA was 143, following Thompson's recommendation of 10 to 20 people per measure.

Continued on next page

Quantitative variables	11	Explain how quantitative variables were handled in the analyses. If applicable, describe which groupings were chosen and why	8-9	The **Statistical analysis** section details how to deal with quantitative variables.
Statistical methods	12	(*a*) Describe all statistical methods, including those used to control for confounding	8-9	Item analysis, exploratory factor analysis (EFA), confirmatory factor analysis (CFA), test-retest reliability analysis, criterion-related validity, and correlation analysis were used in this study
		(*b*) Describe any methods used to examine subgroups and interactions		The two samples were not grouped in accordance with the methodology of this study.
		(*c*) Explain how missing data were addressed		There were no missing data in this study
		(*d*) *Cohort study*—If applicable, explain how loss to follow-up was addressed<br>*Case-control study*—If applicable, explain how matching of cases and controls was addressed<br>*Cross-sectional study*—If applicable, describe analytical methods taking account of sampling strategy	8-9	50 people were randomly selected from the online sample to complete the scale again, and a test-retest reliability analysis was completed based on the results.
		(*e*) Describe any sensitivity analyses		
**Results**				
Participants	13*	(a) Report numbers of individuals at each stage of study—eg numbers potentially eligible, examined for eligibility, confirmed eligible, included in the study, completing follow-up, and analysed	6	A total of 527 questionnaires were received. Finally, 379 samples were included in the analysis, and 148 invalid questionnaires were excluded. Of these samples, 143 were online samples and 236 were offline samples.
		(b) Give reasons for non-participation at each stage		
		(c) Consider use of a flow diagram		
Descriptive data	14*	(a) Give characteristics of study participants (eg demographic, clinical, social) and information on	6	Table 1 shows the detail

		exposures and potential confounders		information of demographics.
		(b) Indicate number of participants with missing data for each variable of interest		
		(c) *Cohort study*—Summarise follow-up time (eg, average and total amount)		
Outcome data	15*	*Cohort study*—Report numbers of outcome events or summary measures over time		
		Case-control study—Report numbers in each exposure category, or summary measures of exposure		
		Cross-sectional study—Report numbers of outcome events or summary measures		The final analyses were conducted on 379 samples, which were primarily comprised of scores on the scales and correlation analyses.
Main results	16	(*a*) Give unadjusted estimates and, if applicable, confounder-adjusted estimates and their precision (eg, 95% confidence interval). Make clear which confounders were adjusted for and why they were included	9-11	The **Results** section presents the main results of this study
		(*b*) Report category boundaries when continuous variables were categorized		
		(*c*) If relevant, consider translating estimates of relative risk into absolute risk for a meaningful time period		

Continued on next page

Other analyses	17	Report other analyses done—eg analyses of subgroups and interactions, and sensitivity analyses		
**Discussion**				
Key results	18	Summarise key results with reference to study objectives	11-13	The Chinese version of the RLI displays satisfactory psychometric properties and loot boxes had a positive correlation with gaming, gambling, anxiety, and depression. .
Limitations	19	Discuss limitations of the study, taking into account sources of potential bias or imprecision. Discuss both direction and magnitude of any potential bias	13	Firstly, this study lacked a credible and sufficient sample of gamblers. Secondly, this study only consisted of participants over the age of 18. Therefore, it’s very necessary to test the validity and reliability of the RLI in adolescents in the future.
Interpretation	20	Give a cautious overall interpretation of results considering objectives, limitations, multiplicity of analyses, results from similar studies, and other relevant evidence	12	More items were retained compared to the original author's scale. The main reason for this difference may be the difference in samples. The samples for this study came directly from video game forums or internet cafes. More than 95% of the respondents had gaming experience, which was higher than the original study (84.8%). Meanwhile, stricter screening criteria were used to ensure the quality of the sample. It can be said that our sample is more suitable for investigating loot box use. In addition, cultural differences may also be a reason. As a new term, ‘loot boxes’ is not yet widely

				used in China. This makes the term 'loot boxes' difficult to understand when translated into Chinese. Although we explained the meaning of 'loot boxes' thoroughly in the questionnaire, and even included pictures of loot boxes in popular online games to help participants understand the term. However, there may still be some participants who do not fully understand the meaning of loot boxes. This cognitive bias may affect the final results of the scale. Overall, the CFA results further showed that the fit indices of the one-factor model met the statistical requirements and were valid across different populations. Thus, the RLI-C has excellent construct validity.
Generalisability	21	Discuss the generalisability (external validity) of the study results	13	The Chinese version of RLI shows excellent reliability and validity, and it can be utilized in the future for the preliminary screening of high-risk populations, providing a reliable theoretical basis for the development and execution of subsequent precise intervention plans.
**Other information**				
Funding	22	Give the source of funding and the role of the funders for the present study and, if applicable, for the		Our study was supported by the

	original study on which the present article is based		Science and Technology Major Special Fund Project of Changsha (No.kh2401006). In which had no role in research design, data collection, analysis or interpretation, manuscript writing, or deciding whether to submit the paper for publication.

*Give information separately for cases and controls in case-control studies and, if applicable, for exposed and unexposed groups in cohort and cross-sectional studies.

Note: An Explanation and Elaboration article discusses each checklist item and gives methodological background and published examples of transparent reporting. The STROBE checklist is best used in conjunction with this article (freely available on the Web sites of PLoS Medicine at http://www.plosmedicine.org/, Annals of Internal Medicine at http://www.annals.org/, and Epidemiology at http://www.epidem.com/). Information on the STROBE Initiative is available at www.strobe-statement.org.
