# Supplementary material for: Psychometric evaluation of the Chinese version of Risky Loot Box Index (RLI) and cross-sectional investigation among gamers of China
Source: PeerJ. 2025 Mar 25;13:e19164. doi: 10.7717/peerj.19164 (PMC11952041; doi:10.7717/peerj.19164)
Supplement: Supplemental Information 5 [file peerj-13-19164-s005.docx]

Two categorical data exist for the raw data and the following are explanations of what the numbers mean:

Q1: Gender

| “1” → “Male” | “2” → “Female” |
| --- | --- |

Q2: Education

| “1” → “Middle school” | “2” → “High school” |
| --- | --- |
| “3” → “Junior college” | “4” → “Undergraduate” |
| “5” → “Postgraduate(Master)” | “6” → “Postgraduate(Doctor)” |
